# The purpose of United Nations Security Council practice: Contesting competence claims in the normative context created by the Responsibility to Protect

**DOI:** 10.1177/1354066116669652

**Published:** 2016-10-07

**Authors:** Jason Ralph, Jess Gifkins

**Affiliations:** University of Leeds, UK and University of Queensland, Australia; Leeds Beckett University, UK and University of Queensland, Australia

**Keywords:** Libya, norms, practice theory, Responsibility to Protect, Syria, United Nations

## Abstract

Practice theory provides important insights into the workings of the Security Council. The contribution is currently limited, however, by the conjecture that practice theory operates on ‘a different analytical plane’ to norm/normative theory. Building on existing critiques, we argue that analysing practices separately from normative positions risks misappropriating competence and reifying practice that is not fit for purpose. This risk is realized in Adler-Nissen and Pouliot’s practice-based account of the Libya crisis. By returning the normative context created by the Responsibility to Protect to the analytical foreground, and by drawing on a pragmatic conception of ‘ethical competence’, we find that pre-reflexive practices uncritically accepted as markers of competence — for example, ‘penholding’ — can contribute to the Council’s failure to act collectively in the face of mass atrocity. Drawing on extensive interview material, we offer an alternative account of the Libya intervention, finding that the practices of the permanent three (France, the UK and the US) did not cultivate the kind of collective consciousness that is required to implement the Responsibility to Protect. This is further illustrated by an account of the Security Council’s failure in Syria, where the permanent three’s insistence on regime change instrumentalized the Council at the expense of Responsibility to Protect-appropriate practice. This changed when elected members became ‘penholders’. Practice theory can facilitate learning processes that help the Council meet its responsibilities, but only through an approach that combines its insights with those of norm/normative theory.

## Introduction

The idea that international society should act collectively through the United Nations (UN) Security Council to protect populations from war crimes, crimes against humanity, ethnic cleansing and genocide remains a normative aspiration. There is evidence to suggest that the aspiration is not utopian. Over recent decades, it has become increasingly likely that the Security Council will not only respond to mass violence, but respond with measures aimed at protecting civilians (Bellamy, 2016). Yet, it is also obvious that the Security Council’s permanent members do not always act collectively. Indeed, the vetoes of the proposed resolutions on Syria demonstrate the level of discord. The purpose of this article is to ask what a focus on Security Council ‘practices’ can tell us about this failure to act collectively. More specifically, it seeks to demonstrate how the recent ‘practice turn’ in International Relations (IR) theory (Adler and Pouliot, 2011a, 2011b; Adler-Nissen, 2013; Adler-Nissen and Pouliot, 2014; Brown, 2012; Bueger and Gadinger, 2014, 2015; Frost, 2009; Frost and Lechner, 2016; Neumann, 2002; Pouliot, 2008; Schindler and Wille, 2015) can inform the process of implementing the Responsibility to Protect (R2P) norm. In addition, the article considers what the normative failures of the Security Council tell us about ‘practice theory’ as it is currently being used in IR.

Practice theory in IR has centred on a definition of practice as the ‘competent performance’ of ‘patterned actions that are embedded in particular organized contexts’ (Adler and Pouliot, 2011a: 6). We accept that this Bourdieu-influenced (Adler-Nissen, 2013) account of practice can make an important contribution to the study of the Security Council and R2P. It does this by foregrounding what is too often in the analytical background. Practice theory, for instance, draws our attention to significant informal Security Council practices, such as ‘penholding’. We argue, however, that the current contribution of practice theory is potentially limited by the conjecture that it operates on ‘a different analytical plane’ to both norm theory and normative theory (Adler-Nissen and Pouliot, 2014: 891; see also Pouliot and Cornut, 2015: 302). Considering practices in isolation from normative positions like that articulated in R2P risks misappropriating ‘competence’ (see Duvall and Chowdhury, 2011: 339) and reifying practice that may not be fit for purpose. Practitioners deemed ‘competent’ by the Bourdieu-influenced approach may actually be making it more difficult to construct and sustain a collective consciousness that responds to mass atrocity in a timely and decisive manner.

The Bourdieu-influenced approach to IR practice theory emphasizes the pre-reflexivity of everyday practice. Other approaches, however, hold a definition of practice that demands diplomats exercise practical judgement on how best to achieve normative goals (Brown, 2012; Frost, 2009; Frost and Lechner, 2016; Hopf, 2010; Kratochwil, 2011). Located in a broader understanding of the ‘pragmatist vocation’ (Abraham and Abramson, 2015), these alternative definitions of practice insist on reflexivity and deliberation so that everyday practices are not impediments to the exercise of good judgement. Practice, in this sense, is a matter of what Mervyn Frost (2009) calls ‘ethical competence’, and it is this, we further argue, that R2P demands of UN diplomats. Diplomats operating in the new normative context created by R2P need to square what they see as the most appropriate response to mass atrocity with the need to construct and sustain a collective cosmopolitan consciousness that underpins the core ethic of protection. Of course, reflecting on the usefulness of fixed definitions of competency and changing the *habitus* surrounding everyday practice may not be enough to enable a consensus that protects vulnerable populations. How exactly states should discharge their responsibility to protect *is* contested. Our argument is merely that ethical competence in the normative context created by a state’s commitment to R2P demands critical self-reflection on the part of the Security Council diplomat so that everyday practice does not become an unnecessary obstacle to formulating a collective response. IR practice theory can encourage this by attributing competence according to broader ethical criteria as opposed to certain professional standards.

To advance this argument, the article is structured in three sections. The first elaborates on Bourdieu-influenced practice theory, outlining the limitations and the risks of separating it from normative theory. The limitations and risks are illustrated in a critical analysis of Rebecca Adler-Nissen and Vincent Pouliot’s (2014) analysis of the North Atlantic Treaty Organization (NATO)-led intervention in Libya. Adler-Nissen and Pouliot demonstrate how Resolution 1973, which authorized the use of force to protect Libya’s civilian population, was influenced by the ‘competence’ in the everyday practices of British, French and US diplomats. Our claim here is that in foregrounding background knowledge, Adler-Nissen and Pouliot have made an overcorrection. Our argument is not that the diplomat’s competence or ‘sense of the game’ is irrelevant to the story. Rather, we claim that the authors attach too much importance to this and that the Council’s normative commitments are shifted too far into the background.

The second section addresses this overcorrection by reintroducing the normative purpose articulated in R2P to the study of the Libyan intervention. Doing this problematizes Bourdieu-inspired practice theory and its application to Security Council practices. This is because those deemed by this approach to be most ‘competent’, the three Western permanent members of the Council (P3), failed to construct an enduring consensus for collective action against mass atrocity. Again, we do not ignore the difficulty of this task, but our criteria of ‘R2P competence’ demands that we assess P3 diplomacy according to its commitment to the R2P norm. This values practices that create and sustain a collective response to mass atrocity. The emphasis here is on the P3 commitment to R2P rather than any claim that the norm is universally accepted. In that context, we find that P3 practice on Libya falls short in two areas: first, against the backdrop of widespread rhetorical support for R2P (Bellamy, 2015; Gifkins, 2016; Welsh, 2013), P3 diplomats still alienated key stakeholders and this weakened the consensus behind Resolution 1973; and, second, while a commitment to ‘regime change’ was not an unreasonable response to the threat that Gaddafi posed, it had a profound impact on the willingness of the Council to trust P3 leadership. In this sense, P3 practitioners demonstrated competency as ‘penholders’ and through their ability to control the international response, but their practices also made the hard task of constructing and sustaining a consensus underpinning R2P even harder. On these grounds, we challenge any claim to ‘ethical competence’.

In the third section, we apply the R2P ethical competence standard to make an assessment of P3 practice during the early stages of the Syrian crisis. Drawing on extensive elite interview data, we focus on Security Council practice surrounding the ‘double vetoes’ in 2011 and 2012, as well as the negotiations in 2014 towards resolutions on humanitarian access.^1^ We argue that P3 competency claims, which underpinned their leadership of the political track during this period, were a substantive and procedural obstacle to the formation of a collective response. Of course, Russia had an interest in protecting the Assad regime and it is therefore right to question its commitment to the R2P agenda. This made the task of negotiating a collective response extremely difficult but that does not mean that we cannot apply the standards of ethical competency to the P3. We argue that ethical competency required the P3 to work with the situation as it was, which meant seeking a resolution that could meet some of the needs of the Syrian population and not let the perfect (regime change) be the enemy of the good (a peace agreement/humanitarian access). We further argue that such a resolution was negotiated but only by taking a fresh look at the competency claims within the Council. When the humanitarian track was taken up by elected members (Australia and Luxembourg, later joined by Jordan — hereafter, the ‘E3’), it became easier to delink humanitarian protection from divisive issues (especially regime change) and to formulate a collective response in line with R2P. This reinforces the central claim, which is that the idea of ‘P3 competence’ is more problematic when we return R2P to the foreground. In this instance, it was E3 leadership that delivered an R2P-appropriate response.

## Security Council practice and the intervention in Libya

### International practice theory

Writing in 2002, Iver Neumann argued that IR theory should return to studying practice (what agents do), as well as text (what agents say). IR researchers had to remind themselves ‘that the linguistic turn and the turn to discourse analysis involved from the beginning a turn to practices’. This meant ‘not just a turn to narrative discourse and rhetoric, but to how politics is actually effected’ (Neumann, 2002: 627; see also Schmidt, 2014). Discourse analysis, Neumann continued, identifies the ‘preconditions’ for social action. It helps the researcher understand how social action is ‘made possible’ (Holland, 2013). However, for Neumann (2002: 628), the analysis is incomplete without ‘the study of social action itself’. It is, he provocatively concluded, ‘armchair analysis’. Without dismissing the usefulness of discourse analysis, this turn to practice can be understood as part of a broader movement towards the classical pragmatism associated especially with William James and John Dewey (Malachowski, 2013; Ralston, 2013). This is interested in ‘useful and reliable’ knowledge that can address really-existing social problems and is not distracted by questions about whether such knowledge is based on ‘incontrovertible’ foundations (Bauer and Brighi, 2009; Friedrichs and Kratochwil, 2009: 702; Kratochwil, 2009, 2011). IR practice theory, in this sense, is a ‘promise to return to the world’ (Abraham and Abramson, 2015: 2).

Neumann thus urged IR researchers to study practices, which he defined as ‘socially recognized forms of activity, done on the basis of what members learn from others, and capable of being done well or badly, correctly or incorrectly’ (Neumann, 2002: 630–631). Forms of activities, like drafting Security Council resolutions, are ‘lived practices’ for state agents, and IR researchers must study them ‘close up’ (Neumann, 2002: 628). Emanuel Adler and Vincent Pouliot responded to this call by developing a practice theory of international relations. Put simply, ‘[p]ractices are competent performances’ (Adler and Pouliot, 2011b: 4). As a performance, individuals (material beings) ‘act out’ a practice; it exists beyond text. A practice is distinguishable from ‘behaviour’ (material doing) and ‘action’ (meaningful behaviour). For Adler and Pouliot (2011b: 5), practices ‘are patterned actions that are embedded in particular organized contexts and, as such, are articulated into specific types of action and are socially developed through learning and training’. To practice in a certain area (e.g. medicine, law, diplomacy) implies competence. Competence, however, ‘is never inherent but attributed in and through social relations’ (Adler and Pouliot, 2011b: 6). As performance, practice implies the existence ‘of an audience able to appraise’ whether it is done correctly or incorrectly (Adler and Pouliot, 2011b: 7). Yet, because practice often involves knowledge that is unspoken (and even unconscious), only those who have practised, and are therefore in receipt of this knowledge, can appraise.^2^ The uninitiated will be unaware of this ‘background knowledge’, and even if they were aware, they would not be well placed to pass judgement on the actions of practitioners. Academics can research practice using an interpretivist approach, which involves, where possible, interviewing practitioners and embedded ethnographic study (Adler-Nissen and Pouliot, 2014; Bueger and Gadinger, 2014; Pouliot, 2013, 2015a; Pouliot and Cornut, 2015). It does not, however, lend itself to normative analysis; a point that is discussed in more detail in the following section.

For Pouliot (2008), ‘background knowledge’ creates a ‘logic of practicality’, which, he argues, can supplement the other logics (of consequences, appropriateness and arguing) that IR theory has concentrated on. Pouliot (2008: 260) argues that these three logics suffer from a ‘representational bias’, that is, they all ‘tend to focus on what agents think about (reflexive and conscious knowledge) at the expense of what they think from (the background know-how that informs practice in an inarticulate fashion)’. Indeed, Pouliot argues that the logic of practicality is ontologically prior to the other three logics because background knowledge makes it possible (or not) to engage in reflexive thinking about consequences, appropriateness and arguing (Pouliot, 2008; Pouliot and Cornut, 2015). Practical, or ‘how to’, knowledge is not only inarticulate and tacit, but ‘pre-intentional’ and ‘pre-reflexive’, and it impacts on the way in which international relations are enacted. Drawing on the work of Pierre Bourdieu, Pouliot argues that background knowledge forms a practitioner’s *habitus*: a ‘system of durable, transposable dispositions, which integrates past experiences and functions at every moment as a matrix of perception, appreciation and action, making possible the accomplishment of infinitely differentiated tasks’ (quoted by Pouliot, 2008: 272) . Relating this to IR, Pouliot suggests that one can talk of a ‘diplomatic habitus’ or, quoting Neumann, ‘a set of regular traits which dispose its bearers to act in a certain way’ (Pouliot, 2008: 272–273).

The insights produced by practice theory are illustrated in Adler-Nissen and Pouliot’s (2014) article on the international intervention in Libya, which is summarized in the following section. Before doing that, however, this section identifies the limitations and risks for practice theory as conceived in this way. Pouliot is clear that practice theory does not aim to make a contribution to normative theory. This is made explicit in his article with Iver Neumann on hysteresis in Russian–Western relations (Neumann and Pouliot, 2011). Hysteresis is another concept drawn from Bourdieu’s work. It describes a mismatch between dispositions and the common-sense rules relevant to social position, and Neumann and Pouliot use it to ‘throw light on the ways in which the clash between Russian and Western diplomatic dispositions have sparked a relentless quest for equal status on Moscow’s part, prompting quixotic practices that are often dismissed by Western countries’ (see also Neumann and Pouliot, 2011: 108; Pouliot, 2010; Schindler and Wille, 2015). This empirical finding is interesting by itself because it reveals the hierarchical and exclusionary dispositions that are reflected in Security Council practice. The point here, however, is what Neumann and Pouliot say (or do not say) about the normative implications of this practice. They write:

our use of the concept of hysteresis is devoid of any normative charge. … We refrain from making the judgment call as to whether Russia is really backward or not. After all, labelling other societies, in particular through hierarchical categories, is an eminently political move that sits uneasily with our analytical objectives. Instead what our article seeks to document is how Western actors, throughout history, have looked down on Russia as a backward society. Hysteresis, thus, is in the eye of the beholder — not out there as an objective or god’s eye reality. There is no absolute grounds from which to assess who is right and who is wrong in a symbolic power struggle. (Neumann and Pouliot, 2011: 114)

Practice theory, in this respect, does nothing to address the charge levelled at constructivism more broadly, which is that it offers an attenuated account of progress and learning (Price, 2008, 2012). The charge is not only that practice theory cannot explain the source of change (Duvall and Chowdhury, 2011; Schindler and Wille, 2015), but also that practice theory has little to say about whether change, when it happens, is progressive. This clearly departs from the classical pragmatist’s reason for focusing on practice, which foregrounds a normative commitment to reflexivity, deliberation, experimentation and democracy as a form of social inquiry (Abraham and Abramson, 2015; Schmidt, 2014).^3^ It is not necessarily a failing, however. It is, in many respects, a consequence of academic practice, an approach to normative and empirical analysis that reflects what Toni Erskine (2012) identifies as a ‘division of labour’: grounded practice theory tells us the *is*, while abstract normative theory tells us the *should*. In this sense, the Bourdieu-inspired practice theory is limited but it is not problematic.

There are, however, normative *risks* to the position taken by Neumann and Pouliot, especially because practice theorists use normatively laden language (e.g. competence) to describe the actions of practitioners. More specifically, by refusing to make a normative ‘judgement call’, practice theory risks co-option by the practitioner who claims competency by virtue of the fact that he or she is the one enacting policy. As noted, the claim that practical knowledge is acquired by doing leaves little (if any) space for external critique. By operating on a single analytical plane — one that notionally separates norms from practice — practice theory can unintentionally reinforce insider values. Indeed, Duvall and Chowdhury (2011: 342–343) warn of potentially ‘pernicious’ consequences if a focus on competent practices ignores those outside the supposed community of practice ‘who contest existing rules using decidedly “incompetent” practices’. Likewise, Chris Brown (2012: 456) warns against the hubris that can accompany the claim to wisdom sometimes made by the experienced insider. This is not to say that insider values are necessarily inappropriate. It does mean, however, that external critique is needed to hold insiders to account. Practice theory is not ‘devoid’ of normativity, even if it seeks to avoid it. As the following section demonstrates, P3 diplomats were considered ‘competent’ because they could dictate agendas, even though, from an R2P perspective, they failed to maintain a collective approach to atrocity prevention.

### The international intervention in Libya

In their article on the 2011 intervention in Libya, Rebecca Adler-Nissen and Vincent Pouliot develop the application of practice theory to IR by demonstrating what they call the ‘emergent power’ that exists in ‘the endogenous resources — social skills or competences — generated within practices’ (Adler-Nissen and Pouliot, 2014: 891). States are able to wield influence when their diplomats cultivate a reputation for competence and skill ‘while undermining similar claims by their opponents’ (Adler-Nissen and Pouliot, 2014: 891). Furthermore, Adler-Nissen and Pouliot explain that the attribute of competence is relational rather than inherent. It has to be cultivated. As such, practice among Security Council members is a ‘struggle for competence’ (Adler-Nissen and Pouliot, 2014). Adler-Nissen and Pouliot’s specific argument is that UK and French diplomats (later joined by US diplomats) were able to dictate the UN response to the Libya crisis not because they necessarily had the best idea on how to respond to the problem, but because they were especially competent in the everyday practices of the Security Council; they had a particularly good ‘feel for the game’ that diplomats play.

Diplomats from these states have certain advantages that translate into social capital. Their state’s permanent membership of the Security Council means that other states respect them as representatives of the Council’s institutional memory. As Adler-Nissen and Pouliot (2014: 898) explain, the UK delegation, with ‘its many seasoned diplomats and numerous “experienced lawyers,” is widely recognized in New York for its superior skills in the many legal technicalities that often bog down the Council’. The UK was, therefore, well placed to take the lead in formulating the UN response to the crisis in Libya. Indeed, the UK is said to have ‘quickly imposed itself as the “penholder” or “lead country” on Libya’, and because its ‘reputation for competence’ was recognized by others, it was ‘easily cashed out in concrete influence’ (Adler-Nissen and Pouliot, 2014: 898). More specifically, in drafting what became Resolution 1970, the British and French were able to transcend opposition to the idea of referring the situation to the International Criminal Court (ICC). As Adler-Nissen and Pouliot (2014) explain, diplomats from these states harnessed the framing power of the media and non-governmental organizations (NGOs) to help them pass the resolution. In this, the P3 were skilled in achieving their objectives.

This performance was quickly repeated with respect to the negotiation of Resolution 1973, which authorized military force to protect Libya’s civilian population. Adler-Nissen and Pouliot (2014) describe a number of practical moves that helped secure the resolution and demonstrate the UK and French diplomats’ expert sense of the game. We are told that their ‘mastery of procedures’ enabled the UK and France to exploit the defection of Libya’s Deputy Permanent Representative (Adler-Nissen and Pouliot, 2014: 899), and that the ‘creative use of UN procedures’ enabled the French to table the British-drafted resolution early, accelerating the negotiating process to prevent the formation of counter-blocs (Adler-Nissen and Pouliot, 2014: 901). To overcome the remaining resistance ‘the P3 resorted to “demarching,” announcing to the Council that “we have a resolution and we go to the capitals to ask for support for it”’ (Adler-Nissen and Pouliot, 2014: 902). Further influence was gained by ‘press harassment’, which involved framing the narrative through media leakages designed to isolate certain states and present them as incompetent (Adler-Nissen and Pouliot, 2014: 902). Resolution 1973 passed with 10 affirmative votes (nine being the minimum) and five abstentions (Brazil, Russia, India, China and Germany). This demonstrated the P3’s success in securing *enough* support, but also demonstrated real divisions, especially in a context where 91% of Council resolutions between 2000 and 2010 passed unanimously (Dunne and Gifkins, 2011: 523). Indeed, without the affirmative votes from South Africa, which came down to the wire, and Nigeria, who said they would vote with South Africa, the resolution would have failed (Adler-Nissen and Pouliot, 2014: 904; see also Cooper and Myers, 2011). This raises questions as to whether a ‘competent’ outcome refers to securing sufficient one-off support or to developing a sustainable collective response.

Our insider interviews confirm Adler-Nissen and Pouliot’s account of the skilled way in which British and French diplomats accelerated the diplomatic process to pass Resolutions 1970 and 1973 (Interviews 2 and 3 (for a list of interviews, see Appendix 1)). As one well-placed source told us, the decision to table resolutions so quickly was perceived by some states as ‘crazy’, even a ‘hostile act’, but the judgement that these resolutions would command sufficient support proved correct (Interview 16). However, there appears to be some confusion in Adler-Nissen and Pouliot’s analysis of this practice, and this has significant normative implications. The confusion stems from the failure to clarify the diplomats’ *purpose*. This follows on from the emphasis placed on foregrounding background knowledge. By representing Security Council negotiations as a struggle *for* competence, the authors imply that the substance of these resolutions and the broader normative context were insignificant. By foregrounding the struggle for power in practice, in other words, the normative purpose of practice is pushed too far into the background and we are left with some odd formulations. The twisting of UN procedures to allow the Libyan defector to speak is represented not as a useful move in passing a resolution that would enable the protection of the Libyan people, but as part of the UK and French ‘struggle for competence’ (Adler-Nissen and Pouliot, 2014: 899). Taking on the Lebanese suggestion to insert ‘no foreign occupation’ was not about ensuring a collective response. It was instead a ‘crafty compromise’, which demonstrated how ‘the successful struggle for competence led to actual influence’ (Adler-Nissen and Pouliot, 2014: 901). As Duvall and Chowdhury (2011: 337) warn, there is an analytical cost to seeing practice as having a ‘univocal meaning’ separate from normative context.

In short, we are left with the impression that practice is not for something (R2P), but for someone (the diplomat). This potential confusion over the purpose of practice is significant because it brings with it the risk of misappropriating (in)competence. Why, for instance, is the German failure to vote for Resolution 1973 represented as an example of its incompetence rather than that of the P3’s? There is a danger here that German doubts about the substance of the resolution (see Brockmeier, 2013), rather than its diplomatic practice, are being labelled incompetent. If this is the case, then the labelling of policy positions as ‘incompetent’ *is* a normative move and practice theory fails to achieve the objectivity it proclaims; indeed, it risks allying itself with the position that the German abstention was ‘shameful’ because it opposed military action. We are told by Adler-Nissen and Pouliot, however, that Germany’s relative isolation was something that its diplomats regretted because it was a consequence of their misreading of the US willingness to vote for the resolution, that is, the abstention was a consequence of bad practice not bad policy. This argument is contested by our interview data, which reinforce Brockmeier’s (2013) point that the abstention was conceived in Berlin not New York (Interview 16). Still, if we accept a practice-based explanation for the abstention, why was the German misunderstanding not a sign of P3 incompetence? They, after all, were trying to garner as many votes as possible for the resolution, as is standard practice in the Council. This is especially the case if we accept that the purpose of P3 diplomacy is not simply the maintenance of their own relative standing within the Council, but to cultivate a sustainable collective response that stops atrocity crimes. On this reading, the vote on Resolution 1973 was not a complete victory for P3 diplomats. The resolution passed but the Council was divided, and the ‘faultline’ would become clearer as the P3 implemented the mandate (Ralph and Gallagher, 2015). The main point here, however, is that this debate exposes the limitation *and* risk of applying practice theory on an analytical plane that is separate to norm and normative theory. If practice theory accepts that the purpose of P3 diplomacy is simply to maintain its diplomats’ reputation for delivering their preferred outcome, and if those that disagree with the P3 are labelled incompetent, then practice theory impedes the kind of reflection that facilitates the process of constructing and maintaining the collective cosmopolitan consciousness underpinning the ethic of protection.

## The purpose of Security Council practice

### R2P competence

According to its critics, practice theory struggles to explain change (Duvall and Chowdhury, 2011; Schindler and Wille, 2015). For their part, Adler-Nissen and Pouliot (2014) accept that practices do change through processes of competence contestation. They offer a thin form of contestation — which happens ‘when the players accept the criteria of competence, but contest the way in which they and others are categorized in order to climb positions in a particular setting’ — and a thick form of contestation — ‘which not only regards the competence of another member, but also involves questioning the definition of competence altogether’ (Adler-Nissen and Pouliot, 2014: 895). In the latter case, a successful form of contestation ‘not only changes the relative position between agents, but the very game itself’ (Adler-Nissen and Pouliot, 2014: 895). The problem with this definition is that it gives no indication of why agents would question the definition of competence, and so long as practice theory is said to operate on a different analytical plane to norm and normative theory, it will not be able to answer that question. The risk here is that the normative challenges to existing practice are not recognized as being significant or legitimate because they operate on a different plane. Of course, the act of foregrounding practice does lead one to assess the practicability of normative ideas, and that is important (Price, 2012), but to do this, one has to make sure that those ideas are themselves in the foreground of one’s analysis.

These risks are realized in Adler-Nissen and Pouliot’s (2014: 895) article when they proclaim that we rarely see a thick form of contestation in the ‘conservative world of multilateral diplomacy’. This is despite the fact that their case study, the intervention in Libya, was described by the UN Secretary General as a situation where the R2P norm ‘came of age’ (Ki-moon, 2012). This norm is often represented as a challenge to the practices associated with sovereignty (Duvall and Chowdhury, 2011), but it is also a challenge to Security Council practices. The 2005 World Summit outcome document, which, for the purpose of this article, is R2P’s reference point, calls on the international community to protect vulnerable populations by taking ‘collective action, in a timely and decisive manner, through the Security Council, in accordance with the Charter, including Chapter VII, on a case-by-case basis’ (United Nations, 2005: 30). This identification of a *collective* Responsibility to Protect stemmed not only from the Council’s failure to intervene to prevent mass atrocity (as in Rwanda), but also from an understanding that if the Council did not act collectively in situations that demanded intervention (like Kosovo), it could lead to a threat to international peace and security. R2P, in other words, *is* a ‘thick’ form of contestation. It is the kind of ‘cosmopolitan or human-centred norm’ that shows ‘diplomacy as we know it … to be a relic’ (Mitzen, 2015: 135).

In seeking to square two potentially competing ends (i.e. human protection and international consensus), R2P demands what Frost (2009) identifies as ‘ethical competence’. As participants of two ‘global practices’, which Frost calls ‘global civil society’ and ‘the society of sovereignty states’, UN diplomats must ‘pay attention to the possible tensions and conflicts between them’ (Frost, 2009: 98). Where conflict does occur, and Frost uses humanitarian intervention as an example, ‘ethical competence’ involves finding ways to ‘harmonize these two practices so that this tension is removed’ (Frost, 2009: 98–99). The ethical skill required here, he concludes, ‘is the ability to achieve ethical coherence across social practices, including the willingness and indeed *competence* to think through and implement practical adjustments’ (Frost, 2009: 99, emphasis in original). A similar view is offered by Chris Brown (2012). He draws a distinction between the Bourdieuian emphasis on *habitus* and an Aristotelian approach that sees practice in terms of *phronesis* (prudence or practical wisdom).^4^ From this perspective ‘the virtuous individual may seem to be acting on the basis of habit but this habitual knowledge is developed consciously through processes of reasoning’ (Brown, 2012: 441). Furthermore, Brown (2012: 441), like Frost, identifies practical skill in ‘the possession of the intellectual ability to think through how things might be different, and to weigh the consequences of action’ (see also Navari, 2011).

There is, then, an alternative to the Bourdieu-influenced definition of practice, and it enables an assessment of Security Council diplomacy in all its normative contexts. Yet, there are also differences within this alternative approach. Frost and Lechner (2016), for instance, critique Brown’s Aristotelian view, arguing that the external ends of the practical judgement exercised by the practitioner are not always clear. This is addressed, they argue, through a Wittgensteinian-inspired definition that sees practice in terms of a language game that helps constitute the rules of international society. From this perspective, the external end of practical judgement is clearer: it is the construction and maintenance of rules that reflect the values of international society. However, in this context, there remains an emphasis on judgement and, drawing on Frost’s earlier concept, practice theory should assess how well diplomats ‘harmonize’ a plurality of values across international society (e.g. human protection and collective decision-making). Of course, the R2P norm tries to do just that, but as Kratochwil (1989) noted, interpreting the meaning of a norm in any given situation is itself a process of practical reasoning. From this perspective, diplomatic performance is, therefore, a matter of ethical competence or practical reasoning in the context of the norms that states are committed to and not simply professional competence in the context of diplomatic *habitus*. More specifically, in the normative context created by states’ commitments to R2P, ethical competence requires responding to mass atrocities in ways that build and maintain a cosmopolitan consciousness.

Assessing the performance of Security Council diplomats in these terms, which we call R2P competence, does not necessarily mean that one will reach different conclusions to Adler-Nissen and Pouliot. The British and French diplomats negotiating Resolutions 1970 and 1973 can be described in the context created by their commitment to R2P as somewhat competent. They delivered a workable consensus on how to respond to the situation in Libya, but our argument is that it was weak and unsustainable. Much has been written, for instance, on the claim that the Council did not sign up for regime change in Libya and that P3 actions were therefore a betrayal of the Resolution 1973 mandate (see, e.g., Cronogue, 2012; Morris, 2013). Our interviews with practitioners who negotiated that resolution stress the importance here of US Ambassador Rice being extremely clear before the Council: military action would be taken against Gaddafi’s regime and not restricted to a limited defence of Benghazi (Interviews 5 and 16; see also Glasser, 2012). From this perspective, those that complained about the extent of the military operation were being ‘disingenuous’. Yet, these accounts of Rice’s clarity are told as if they justify the closing down of a diplomatic process after the negotiation of Resolution 1973, which, from the perspective of other interviewees, they do not (Interview 4).

First, it was extremely difficult for any state which agreed that military action was needed to defend Benghazi to withdraw support for Resolution 1973 on grounds that the NATO-led military action outlined by Rice would go too far. There was only one military option available. The sceptics had little diplomatic room for manoeuvre given the imminent threat of atrocities. Second, when states such as South Africa did seek input into the execution of the military option, they were told in no uncertain terms that their efforts were unwelcome. Despite the fact that operative paragraph 2 of Resolution 1973 called for full implementation of the negotiating process created by the African Union ‘road map’, a process that could have been pursued without prejudicing the defence of Benghazi, the P3 made it clear that this would not happen. We were told by one diplomat, for instance, that the mediation team preparing to go to Libya was warned by France not to travel because the bombing was about to start (Interview 4). As another diplomat put it, the P3 had little time for what they saw as ‘a deeply misguided’ mediation effort involving ‘some tent in the desert’; it flew in the face of everything they knew about the Gaddafi regime (Interview 5). Another member of the elected 10 (E10) said that they wanted a panel of experts to report back to the Council on the implementation of Resolution 1973, which may have helped to facilitate ongoing dialogue (Interview 18).

Putting this in the context of Adler-Nissen and Pouliot’s analysis, one might again argue that it demonstrates P3 competence. However, if we reintroduce the importance of collective decision-making to norm implementation, an interpretation of Resolution 1973 that leaned towards regime change puts that analysis in a different light. Indeed, the alienation of some Council members was exacerbated as the military mission continued beyond the expected time frame. Delays in NATO reporting to the Council — the first was delivered in April — compounded the frustration.^5^ Perhaps the most problematic move, however, was the creation of the Libya Contact Group at the London Conference of 29 March 2011. The Group was made up of the P3 and representatives from the League of Arab States, the Organization of Islamic Cooperation, the Gulf Cooperation Council, the European Union and NATO; while Russia and China were invited, it was clear that they would not attend given their perception that the Group undermined the Security Council and that it was working towards an end — regime change — that, for them, went beyond the civilian protection mandate (Allison, 2013: 197). For its part, the P3 never considered returning to the Council to renew the legitimacy of the mandate. This is because the circumstances surrounding the passage were considered exceptional and that returning to the Council would result in deadlock. This judgement did not avoid negative consequences, however. By acknowledging that political support for the P3 had changed since Resolution 1973 had been passed, but insisting that the way in which NATO was implementing the mandate was correct, P3 diplomacy had seemingly given up on trying to harmonize human protection and collective decision-making.

It might be argued that the Libya Contact Group’s membership *was* sufficiently inclusive, and that this bestowed legitimacy even in Russia’s and China’s absence. That is not an unreasonable position, especially if it can be shown to have delivered good outcomes, which, of course, is not necessarily so in this particular case. R2P, however, requires the cultivation of a collective cosmopolitan consciousness *at the UN Security Council*, and, in this respect, the P3’s diplomatic competence was misdirected. The P3 could clearly assemble a large international coalition, but it did not give sufficient weight to the opinions of Russia and China (known as the P2) and this was not without consequence for the perpetuation of a collective consciousness based on atrocity prevention. In fact, the creation of the Contact Group reinforced a narrative within Russia that the cooperative arrangements it had with NATO were meaningless and that the ‘Libya model’ had revealed NATO ambitions to encourage and exploit instability on Russia’s periphery (Allison, 2013: 195). The idea of working through the NATO–Russia Council to supplement UN diplomacy on Libya was raised but only on the margins of P3 discourse (Owen, 2012). As we shall see in the following, this continuing sense of exclusion informed Russia’s approach to the Syria situation, where the P3 pursued a similar approach through the informal Friends of Syria grouping.

The argument here, then, is that the R2P norm, and the emphasis it places on the Security Council’s collective responsibility to prevent atrocities, *does* pose a challenge to the ‘conservative world of multilateral diplomacy’ and the definition of competent practice that goes with it. Competent diplomacy in this new normative context is about cultivating and sustaining a collective Security Council consciousness based on the principle of protection. It is not simply about dictating the agenda so that one’s preferred substantive outcome is pursued in a particular case. Indeed, Pouliot (2015b; see also Mitzen, 2015; Sending, 2015) seemingly acknowledges this when he argues that permanent representatives at international organizations (like the UN) see themselves as responsible for global governance, as well as for securing the national interest. Implicit in this higher competency standard, however, is a potential dilemma: what if the compromises made to facilitate a collective response produce outcomes that are not consistent with the principle of protection? Can this be considered competent R2P diplomacy? Vice versa, what if acts intended to protect populations do not command the support of the P5 at the Security Council? Is their pursuit consistent with competent R2P practice? The need to act against this background reminds us of the enduring importance of context-sensitive judgement, as well as the need to reflect more generally on R2P’s meanings so that its practices do not become fixated by what appears to be an insurmountable dilemma.

### The practice of penholding

A practice that has come under increasing criticism from Security Council practitioners, particularly in response to Syria, is ‘penholding’. A ‘penholder’ is a Council member who initiates and chairs the informal process of drafting a resolution, and the practice took on new meaning in 2008 as a response to the demands of the Council’s growing agenda (Security Council Report, 2013; Sievers and Daws, 2014: 267). While the practice was never meant to indicate ‘political ownership’, it has become more structured, with one state taking the lead on all resolutions relating to a given topic. The level of ownership is such that if the penholder does not initiate a draft, the agenda item sits dormant (Interview 2). In theory, this role could rotate between Council members. In practice, however, the majority of country-specific agenda items have been divided between the P3, giving them increased dominance over the drafting process (Security Council Report, 2013; Tardy and Zaum, 2016). This limits the level of input that non-P3 members can have in negotiations, and particularly marginalizes elected members of the Council.

As a consequence of this practice, the P3 have been able, with increasing frequency, to set the terms of debate. The penholder practice enables them to frame an issue and lay out a course of action before consulting others. As described by Germany’s former Permanent Representative to the United Nations: ‘The one who leads, the one who presents the text, who stakes out a position early in the day, is the one who more or less determines the game’ (quoted in Lynch, 2014). Once a draft has been prepared, it is then commonly circulated and refined between permanent members before being shared with elected members. At that stage, however, there is often little scope for further input. Our interview data reveal much frustration with the penholding practice.

While, for Adler-Nissen and Pouliot, this practice was a mark of competence, for practitioners among the E10 states, penholding is a practice that weakens deliberation; it has to be endured rather than embraced. These interviewees reported limited time for consultation once elected members are privy to the text, a sense that deadlines were manufactured to close down discussion and that the role of elected members was often to ‘rubber-stamp’ decisions that had already been made in negotiations between permanent members (Interviews 10, 14 and 18; see also Keating, 2016; Niemetz, 2015; Security Council Report, 2014; Wenaweser, 2016). The collapse of the fragile consensus that the P3 created on Resolution 1973 must be understood in this context. The criticism of the manner in which the P3 implemented Resolution 1973 drew intensity from a sense that it was symptomatic of the exclusionary hierarchies that are deeply ingrained within Security Council practices.

There have been attempts to reform these hierarchical practices through annual meetings of the Council’s group on working methods. However, a sense of exclusion persists and this is not outcome-neutral. For instance, Argentina’s position on the 2014 French draft resolution referring the Syria situation to the ICC was influenced by its frustration at the lack of Security Council reform (Ralph, 2016). The claim here is not that P3 compromise and the democratization of these practices would necessarily lead to a collective response to the problem of mass atrocity. The claim is more modest. It is simply that the practices that Adler-Nissen and Pouliot (2014) describe as marks of competence are, in fact, problematic when we reintroduce a normative standard that values an ability to cultivate and sustain a collective consciousness. Competent R2P diplomacy is not simply marked by an ability to secure one’s preferences. As the following section demonstrates, negotiating an R2P-appropriate resolution was made more difficult by P3 leadership, which insisted on prioritizing the political track even in the context of mistrust created by its Libya intervention. An R2P-appropriate resolution was eventually negotiated, but under E3 leadership. Changing the penholder enabled a pragmatic approach that was focused on solving the problem of humanitarian access in the context of broader political disagreement.

## Security Council practice and the Syria crisis

### The failure to respond collectively

Russia and China obviously bear responsibility for the vetoes of the P3 draft resolutions on Syria. It does not follow, however, that the P3 were ethically competent in the normative context created by their commitment to R2P. Scrutinizing P3 judgement when drafting those resolutions is important because it helps make the central point: the definition of competence that Adler-Nissen and Pouliot use to assess state practice at the Security Council is incomplete once the R2P norm is returned to the analytical foreground. A focus on the normative context created by the P3’s commitment to R2P problematizes Western insistence on regime change, as articulated in the ‘Assad must go’ refrain. Of course, it was not unreasonable for the P3 to argue, as they had done on Libya, for the regime to be replaced. However, that argument failed to give adequate weight to various factors that made it unrealizable. These factors include the post-Libya lack of trust at the Security Council, Russian and Iranian interests in protecting Assad, and an unwillingness to embark on direct or indirect military intervention, which was necessary to overthrow the regime. We argue in this final section that by calling for Assad to go, P3 practice lacked sound judgement in terms of what was practically possible. It may have helped to construct an image of the Western states being ‘on the right side of history’, but it also contributed to the Security Council’s failure to agree on practical ways of protecting the Syrian population.^6^ It was not R2P-competent practice.

At the beginning of October 2011, the UK tabled a resolution that condemned the Assad regime and called for sanctions under Article 41 of the UN Charter. Whether the Russians would have vetoed this resolution in the absence of the discord created by the Libya intervention is a matter of contention. Although Russia has made forceful statements linking its position on Syria to the P3’s overreach on Libya, it has been inconsistent in linking the two cases, and Russia’s position can also be explained by other factors, such as broader resistance to external regime change and bolstering an image of power both at home and abroad (Allison, 2013; Bellamy, 2014; Gifkins, 2012). Russia’s long-standing alliance with the Assad regime and its material interests in that country suggest that the veto was inevitable. This line of argument stresses that P3 draft resolutions were not a prelude to military action for the purpose of overthrowing the Assad regime. By claiming otherwise, the Russians were using a misplaced concern among the elected members to protect their particular interests.

It is clear, both from a textual analysis of what the Russians said (United Nations, 2011: 3–5) and from our interview data (Interviews 4 and 18), however, that P3 practice on Libya and the frustration it caused within the wider Council made it easier for Russia to cast its veto. China joined Russia in voting against; Brazil, India, Lebanon and South Africa abstained.^7^ The rationale for these votes was that the P3 should not be allowed to repeat at the Security Council what it had done earlier in the year with respect to Libya. Moreover, Western governments had called on Assad to go and the possibility of military intervention (especially by Turkey) was a consistent feature of Western discourse. Indeed, our interview data suggest that there was a view within the P3 that the diplomatic practice surrounding Resolutions 1970 and 1973 could and should be repeated. As one well-placed diplomat put it (Interview 16), there was a view that ‘the Libya experience had been a good exercise. There was a sense of being all-powerful’. The Syria situation was different, but there remained a sense that a Libya-type diplomatic process could deter attacks on Syria’s population. From this perspective, P2 concerns ‘that they were seeing the same methods as were deployed during the process of negotiating the Libya resolutions’ and that the Security Council was ‘being turned into a machine for regime change’ (Interview 18) were not entirely misplaced.

This is an interesting debate and it has some relevance to an assessment of R2P competence, but it is not decisive. The motivation behind the ‘double vetoes’ is less relevant than the fact that Russia and China opposed regime change in Syria. In this context, any P3 proposal that linked human protection to regime change was not R2P-competent because it could not be squared with the norm’s insistence that the response to mass atrocity be coordinated through the Security Council. By not accepting that, P3 diplomats made an error of judgement on what was practically possible. Furthermore, there are grounds for arguing, with some qualification, that P3 diplomats were more interested in claiming the moral high ground than in negotiating a collective response (Interviews 2, 3, 4, 5, 7 and 14). This may not have been the case in February 2012 when Russian Ambassador Vitaly Churkin surprised the Council by vetoing the draft resolution on the morning of the vote. Here, the P3 may be forgiven for thinking that they had done enough to secure consensus and force Assad to recalculate. Yet, in October 2011, July 2012 and May 2014, the P3 continued to pursue their own preferred response while knowing that a draft resolution would be vetoed. This enabled Western governments to put themselves on what they saw as the right side of history and to portray the P2 as obstacles to progress (Glasser, 2012).

Of course, it might be argued that such a strategy was not exclusively concerned with protecting the self-images of the P3. It can also be understood as an attempt to shame Russia into changing its position. P3 rhetoric following the vetoes suggests that such a strategy was in operation. The US said that it was ‘disgusted that a couple of members of this Council continue to prevent us from fulfilling our sole purpose here’ (United Nations, 2012: 5). The language from the UK was even stronger and more direct, stating that it was ‘appalled by the decision of Russia and China to veto an otherwise consensus resolution’, and it continued to say that Russia and China ‘have failed in their responsibility as permanent members of the Security Council’ (United Nations, 2012: 6–7). Pointed public criticism like this is rare within the Council chambers, and there are good arguments in favour of states using shaming as an alternative to more coercive measures (Pattison, 2015). If, however, the intention was to change Russian and Chinese defence of the Assad regime, the strategy failed. On this occasion, and in the absence of alternative courses of action that could have effected the P3’s preferred policy of regime, change, one can question the practical judgement behind the shaming strategy. If the only means of protecting the Syrian people was to work through the Security Council, then alienating key members by shaming them was not prudent diplomacy.

### Changing the penholder: The role of the elected member

In this particular situation, our definition of R2P competence required an alternative approach to harmonizing human protection and collective action. The so-called ‘humanitarian track’ offered such an alternative. This sought to negotiate humanitarian access to vulnerable populations, and while it was not the ideal way for the Council to discharge its protection responsibilities, it was less contentious and, therefore, more realizable than the insistence on regime change. The Russians and Chinese, for instance, did not protest when the UK/Morocco draft resolution of February 2012 called for humanitarian access or when resolutions were limited to supporting confidence-building measures with UN observer missions.^8^ Yet, Security Council resolutions enabling humanitarian access were only passed in 2014. Two factors facilitated the negotiation of these resolutions: a change in political circumstances and a change of penholder. First, in September 2013, the P3 had failed to win public support for a military response to the chemical weapons attack, and that removed the Libya-type scenario from all plausible consideration. In addition, the atmosphere within the Council improved markedly when the P3 decided to follow Russia’s plan to peacefully remove chemical weapons from the Syrian battlefield. Second, a change of penholder in New York enabled the Council to act on this changed political context. More specifically, it was significant that the penholders came from the E10 membership. Our interviews suggest that their status as non-P3 members enabled them to better exploit the new situation and to deliver a collective response to the humanitarian crisis. This is further evidence that the attribution of competence to P3 practice should not go unchallenged once the R2P norm is reintroduced to the analytical foreground.

When Australia picked up the pen on the issue of humanitarian access, it was greeted with scepticism by the P5 (Interview 2). This scepticism appeared justified in July 2013 when negotiations stalled. In October 2013, however, Australia, which had by then been joined by Luxembourg, found sufficient consensus for a presidential statement. This reinforces the earlier claim that simply changing the penholder was not the only factor enabling a collective response. The month following the resolution of the chemical weapons issue was described to us as a ‘perfect moment’ to push on humanitarian access (Interview 1), which illustrates the importance of practitioners understanding timing and context as part of competent practice. Nonetheless, Australia and Luxembourg were not pushing at an open door. The modalities of the delivery of humanitarian assistance (what, by whom, where, via what routes, with whose authority, under what monitoring) were highly contentious. For instance, Russia objected to language that was obviously directed at the government forces. The E3 took the view, however, that a resolution without such language would not address the practical question of how to relieve the humanitarian situation. The political context may have changed but creative diplomatic practice was still required of the E3.

The E3 also had to deal with concerns that the humanitarian initiative would complicate the political track, which had been revived as the Geneva II talks. The P3, in particular, worried that the E3 would concede too much to the Russian position and they would find themselves outmanoeuvred. It is testament to the negotiating skill of the E3 diplomats that they were able to walk this thin line and avoid alienating either the P3 or P2. In fact, they were able to convince the P2 that a resolution on humanitarian access was not part of a Libya-type strategy that would ultimately lead to military-enforced regime change, while still being described by one interviewee as ‘fantastic partners’ to the P3 (Interview 5). Inviting Jordan to join them when it entered the Council in 2014 was also important. Not only was Jordan the Arab representative on the Council, but it was bearing much of the humanitarian burden by hosting many Syrian refugees. It added a moral weight and legitimacy to the argument that Australia and Luxembourg were making, as did the demarches of other Arab states (Interviews 13 and 14).

Finally, insider interviews emphasize the significance of the pragmatic approach adopted by the E3 penholders. Their focus was very clearly on the needs of the Office for the Coordination of Humanitarian Affairs (OCHA) rather than on ‘point scoring’ to secure ‘a victory for one side or another’. Negotiations were ‘not about political differences on the nature of conflict … but about practical solutions’ (Interview 1; see also Interviews 7, 13 and 14). Close observers of the Council reinforce this view, arguing that E3 leadership was very important in overcoming what was a very real P3–P2 divide. From this perspective, the resolution would not have passed had the pen remained in the hands of the P3 (Interviews 1, 2 and 11: see also Langmore and Farrall, 2016). This is telling because it resonates with the definition of competence that informs Adler-Nissen and Pouliot’s conception of practice and P3 performance. In that account, competence is equated with ‘victory’ in the struggle to determine the agenda. The argument here, however, is that when an ethical/R2P lens is imposed on the assessment of Security Council practice, the attribution of diplomatic competence needs redirecting.

Resolution 2139 passed in February 2014, demanding humanitarian access in Syria. It was tabled to coincide with the Sochi Olympics in the hope that this would make it more difficult for Russia, in particular, to cast its veto.^9^ It represented a breakthrough in finding a collective response that was R2P-appropriate, although doubts remained concerning its ability to effect material change on the ground. Regular reports to the Council from OCHA noted a lack of implementation of Resolution 2139 and a deteriorating humanitarian situation. In response, the E3 pushed for another resolution that authorized UN humanitarian agencies and their implementing partners to use routes across conflict lines and border crossings without the consent of the Syrian government; again, the E3 demonstrated a pragmatic commitment to practical problem solving that, to the surprise of some Council insiders, enabled the passage of Resolution 2165 in July 2014. This was significant in the context of P2 suspicion of R2P because it decided humanitarian agencies could cross conflict lines *without Syrian consent*. It appears that what made it difficult for the Russians and the Chinese to veto Resolution 2165 was the E3’s exclusive focus on humanitarianism. While the resolution conditioned Syrian sovereignty, it did so for reasons that could not be linked to political interference; it only referenced the political situation to emphasize ‘that the humanitarian situation would continue to deteriorate further in the absence of a political solution to the crisis’ and to pay tribute to the UN Special Envoys Lakhdar Brahimi and Staffan de Mistura. In other words, an R2P-appropriate resolution was passed by the Security Council, but only because it delinked humanitarianism from regime change and because E3 (as opposed to P3) leadership facilitated a pragmatic rather than ideological approach to the problem. Again, this conclusion challenges the attribution of competence set out in Adler-Nissen and Pouliot’s account of Security Council practice.

## Conclusion

Our analysis shows that there are significant barriers to R2P competence within current Security Council practices. Analysing Security Council practices towards the Libyan case without a normative frame led Adler-Nissen and Pouliot to conclude P3 competency, while adding an R2P lens challenges that conclusion. The difficulty of building and sustaining a consensus on how to respond to situations involving mass atrocity should not be underestimated, but that does not mean that P3 practice should escape scrutiny. Indeed, their own commitment to R2P means that their practice should be assessed according to the ethical standards embedded in that norm. Reintroducing norm and normative theory in this way foregrounds the question ‘What is competency for?’ By taking P3 commitment to R2P as the starting point for analysing practices, the standard of competence shifts from the ability to achieve desired goals to the ability to cultivate and sustain a collective consciousness towards the protection of civilians. Practices such as P3-dominated penholding exacerbate the difficulty of developing a collective consciousness and approach. These exclusionary practices have real implications for how the Council responds to specific cases. While the P3 were unable to make progress on the political and humanitarian tracks on Syria, for instance, the E3 were able to achieve a measure of progress in responding to the humanitarian crisis in Syria.

Although E3 penholding on Syria’s humanitarian track was more able to act creatively to circumvent the deadlock, it has faced real challenges. Indeed, when Australia and Luxembourg finished their terms on the Council, the continuity of Jordan was crucial for continued E10 leadership on Syria’s humanitarian track. The quick manoeuvring of Spain and New Zealand to take their places as E3 members prevented the P3 from reasserting control of penholding (Interview 2). The P3 remain resistant to innovations in penholding that dilute their control over the drafting process (Niemetz, 2015: 115–116). Penholding and other exclusionary practices limit the full involvement of all Council members and thus the capacity for the full development of a collective consciousness in the Council. In this context, and as our argument highlights, there is a risk that the Bourdieu-influenced practice turn in IR theory will simply reify a practice like penholding and leave unchallenged the attribution of competence that goes with it. Fortunately, this risk is mitigated by an alternative conception of practice. This equates diplomatic competence with the exercise of practical judgement that harmonizes a plurality of normative commitments. It is this alternative approach that has been applied in this article, which found that P3 practice fell short of what R2P demands.

## Notes

1. To access Security Council practices, we conducted 18 in-depth interviews with individuals who were either participants in the drafting of resolutions on Libya and Syria, or who closely observed participants. These interviews included staff from UN permanent missions who served on the Security Council at the time, UN Secretariat staff and non-governmental organizations (NGOs) focusing on UN diplomatic negotiations in New York. Not all interviews are cited here. Interview material was triangulated with public news sources and detailed accounts of negotiations from the think-tank Security Council Report. Most interviewees requested that their names, titles and states were not to be disclosed, so Appendix 1 lists anonymized interview information.

2. On the ‘conventional wisdom’ that diplomats ‘see themselves as a single, hermetically sealed, professional community’, see Wiseman (2015: 326). On the ‘suaveness’ and ‘urbane style’ of this community, something that cannot be taught, but ‘is best acquired through upbringing’, see Kuus (2015: 373). The exclusionary implications are obvious.

3. Others demonstrate how a Bourdieu-inspired definition of practice, which includes theory *as* practice, can highlight exclusionary and hierarchical relationships without explicitly denying a normative position, arguing instead that it forms the basis of a reflexive approach to IR (see Berling, 2012; Eagleton-Pierce, 2011; Hamati-Atayi, 2012). For discussion and critique, see Knafo (2016), and for an argument that Bourdieuian reflexivity is a limited ‘politics of exposure’, which is a diminished form of pragmatism, see Abraham and Abramson (2015).

4. See also Hopf (2010: 6), who notes that ‘the logic of practice is more reflective and agential than the logic of habit’, and Kratochwil (2011: 47–48), who sees practice as performance and judgement rather than habit.

5. The frustration was with the nature, as well as the timing, of the reporting. The reports of those engaged in military action were not convincing and lacked credibility. A more thorough and impartial reporting would have involved the UN Secretariat (Interviews 4 and 18).

6. Stephen Walt (2016) makes this point in an argument that clearly resonates with Brown’s (2012) Aristotelian definition of practice and its appearance in the classical realist thinking of, for example, Morgenthau and Kennan. Critiquing US practice (but it applies equally to the P3 as a whole), Walt writes: ‘Obama declared “Assad must go” in Syria despite there being no good way to ensure his departure and no good candidates to replace him, *and then the United States helped block the initial UN efforts to reach a cease-fire to end the fighting*’ (Walt, 2016: emphasis added).

7. While the Chinese were frustrated with the manner in which Resolution 1973 was implemented, their position on Syria owed much to the fact that the Syrian delegation at the UN remained loyal to the regime and was not calling for intervention. The fact that their Libyan counterparts had defected to the rebels allowed the Chinese to square the call for intervention in Resolution 1973 with the principle of sovereign consent (Interview 10).

8. For instance, Resolution 2043, passed unanimously on 21 April 2012, established the UN Supervision Mission in Syria.

9. Interviewees mentioned this deliberate timing but remained unsure as to whether this had an effect on P2 decision-making (Interviews 11 and 14).
